# miR-17-3p Exacerbates Oxidative Damage in Human Retinal Pigment Epithelial Cells

**DOI:** 10.1371/journal.pone.0160887

**Published:** 2016-08-09

**Authors:** Bo Tian, Daniel E. Maidana, Bernard Dib, John B. Miller, Peggy Bouzika, Joan W. Miller, Demetrios G. Vavvas, Haijiang Lin

**Affiliations:** 1 Retina Service, Angiogenesis Laboratory, Department of Ophthalmology, Massachusetts Eye and Ear Infirmary, Harvard Medical School, Boston, MA, United States of America; 2 Department of Ophthalmology, The First Affiliated Hospital of Zhengzhou University, Zhengzhou, Henan, P.R. China; Medical University of South Carolina, UNITED STATES

## Abstract

Oxidative stress has been shown to contribute to the development of age-related macular degeneration (AMD). MicroRNAs (miRNA) are small non-coding RNA molecules that function in RNA silencing and post-transcriptional regulation of gene expression. We showed miR-17-3p to be elevated in macular RPE cells from AMD patients and in ARPE-19 cells under oxidative stress. Transfection of miR-17-3p mimic in ARPE-19 induced cell death and exacerbated oxidative lethality that was alleviated by miR-17-3p inhibitor. The expression of antioxidant enzymes manganese superoxide dismutase (MnSOD) and thioredoxin reductase-2 (TrxR_2_) were suppressed by miR-17-3p mimic and reversed by miR-17-3p inhibitor. These results suggest miR-17-3p aggravates oxidative damage-induced cell death in human RPE cells, while miR-17-3p inhibitor acts as a potential protector against oxidative stress by regulating the expression of antioxidant enzymes.

## Introduction

Age-related macular degeneration is the leading cause of irreversible blindness among the elderly in developed countries. Although therapeutic strategies for the less prevalent neovascular form exist, therapies for the more prevalent non-neovascular (“dry”) form are lacking. A better understanding its pathogenesis may guide researchers towards the development of novel therapies. It has been postulated that oxidative stress [1–4] and inflammation [5–13] play a critical role in AMD pathogenesis. Retinal pigment epithelium (RPE) cells are extremely important to photoreceptor function, being responsible for the recycling of the visual pigments and in phagocytosis of photoreceptor outer segments [14,15]. Due to its location in a highly oxygenated and lit environment, RPE is at high risk for oxidative injury that could lead to cellular dysfunction, inflammation, and eventually cell death [2–4,16–20].

There are several enzymes responsible for oxidative stress management, such as superoxide dismutase (SOD) and thioredoxin reductase. Among the three SOD isoforms, MnSOD is the essential mitochondrial antioxidant enzyme. It has been demonstrated that RPE cells deriving from MnSOD-deficient mice are more susceptible to oxidative stress than wild-type RPE cells [21]. Three well characterized isoenzymes of thioredoxin reductases, namely cytosolic TrxR_1_, mitochondrial TrxR_2_, and testicular TrxR_3_ are flavoproteins that reduce thioredoxin, a major protein involved in the reduction of cellular oxidative stress[22,23]. TrxR_2_ controls H_2_O_2_ emission by maintaining the level of active thioredoxin [24], which has been show efficiently decreased RPE cell death caused by oxidative stress [25]. Overall, dysfunction of antioxidant enzymes can cause ROS accumulation, and ROS defense systems have been shown to be important in RPE [26–36]. Consequently, the regulation of antioxidant enzymes is important in the study of AMD, as efficient antioxidant defense systems are needed to protect the RPE cells.

MicroRNAs (miRNAs) are small non-coding RNAs that play an essential role in regulating gene expression, either by degrading messenger RNA (mRNA) or stalling translation [37]. They can be generated from either the 5p or 3p or both arms of pre-miRNA, and depending on which arm they are generated from, they are notated as: miR-#-5p or miR-#-3p [38]. These molecules have been proven to be involved in extensive pathological processes, including angiogenesis, oxidative stress, immune response and inflammation [39–42], all of which are critical processes in age-related macular degeneration (AMD). MicroRNA-30b has previously been shown to impair oxidative stress mechanisms in ARPE-19 [43], whereas miR-9 has been shown to be upregulated by a retinoic acid analogue in the same cells [44]. Using human iPSC-derived RPE cells under Paraquat stress, Garcia et al. showed upregulation of miR-146a and miR-29a, downregulation of miR-144, miR-200a and miR-21, whereas a biphasic response was seen on miR-27b [45]. We previously reported that miR-23 enhances RPE cell resistance to oxidative stress damage and is downregulated in macular RPE cells from AMD patients [46]. miR-17-3p is a member of miR-17/92 cluster, originally found to be involved in tumorigenesis, but more recently, members of this cluster have been shown to be involved in many aging disorders [47]. Although most of the work regarding miR17-3p has focused on regulation of cell proliferation pathways, a study using prostate cancer cell lines demonstrated that miR-17-3p is also involved in regulating antioxidant enzymes [48]. In this study, we aim to explore the role of miR-17-3p in ARPE-19 cell viability and antioxidant enzyme production under oxidative stress, a major factor in AMD pathogenesis.

## Materials and Methods

### Cell culture

Human donor eyes from AMD patients (70–90 years old) and age-matched controls were acquired from the Minnesota Lions Eye Bank (Saint Paul, MN), in accordance with the provisions of the Declaration of Helsinki for research involving human tissues. Macular RPE cells were isolated as explained previously [49]. Briefly, cornea, anterior segment, vitreous, and neural retina were carefully removed without disturbing the RPE layer. The dissection was performed by an 8-mm sterile trephine punch through the RPE cell layer, Bruch’s membrane, and choroid, centered on the macula. RPE cells from this region were collected as macular RPE cells. Subsequently, the RPE cells were dissociated after trypsin digestion (30 minutes at 37°C) in pre-warmed medium (DMEM/F12; Cat#11330–057, Gibco, Grand Island, NY). Centrifugation of cells for 5 minutes was carried at 168 × g at 4°C. Supernatant was carefully aspirated and the cell pellet was re-suspended in DMEM medium. To guarantee the purity of RPE cells during the isolating procedure, the isolated cells were analyzed morphologically and stained with cytokeratin-18 antibody, which confirmed that the percentage of RPE cells was more than 97%.

Human retinal pigment epithelium and ARPE-19 cells (CRL-2302, ATCC, Manassas, VA) were cultured in medium (DMEM/F-12; Cat#11330–057, Gibco, Grand Island, NY) supplemented with 10% fetal bovine serum (FBS; Cat#10438–026, Gibco, Grand Island, NY) and penicillin-streptomycin (100 U/mL-100μg/ml; Cat#15140, Gibco, Grand Island, NY) at 37°C in a humidified 5% CO_2_ incubator.

### miRNA transfection

ARPE-19 cells were cultured in DMEM/F12 medium to reach 70% confluence, and transfection was performed for functional analysis. miR-17-3p mimic (Cat#4464066, Life technologies), antisense miR-17-3p mimic (inhibitor, Cat#4464084, Life technologies), scrambled mimic (sc-mimic, Cat#4464058, Life technologies), and scrambled inhibitor (sc-inhibitor, Cat#4464076, Life technologies) were transfected into ARPE-19 cells with Lipofectamine® RNAiMAX (Cat#13778030, Invitrogen, Carlsbad, CA) according to the manufacturer’s manual, respectively. The final concentration of miRNAs was 20 nM. Cells were then incubated for 72 h in a humidified incubator with 5% CO_2_ at 37°C until utilized for experiments.

### H_2_O_2_ and TBH treatment

ARPE-19 cells were seeded to a density of 3.5 ×10^5^/ml in 6-well plates, and cultured in medium supplemented with 10% fetal bovine serum and penicillin-streptomycin. Twenty-four hours later, cells at 90~100% confluence were exposed to H_2_O_2_ (25, 50, 100, or 200μM) (Cat#H-1009, Sigma-Aldrich, St.Louis, MO) and tert-Butyl hydrogen peroxide solution, TBH (Cat#458139, 37.5, 75, or 150μM) (Sigma-Aldrich, St. Louis, MO) diluted in cell culture medium (DMEM/F12; Cat#11330–057, Gibco, Grand Island, NY) without fetal bovine serum or phenol red.

### Cell viability assay

Cell viability was determined by 3-(4,5-dimethylthiazol-2-yl)-2,5-diphenylt-etrazolium bromide (MTT, Cat#M6494, Molecular Probes, Eugene, OR) assay. Specifically, after the incubation, the cell medium was aspirated from the 96-well plate. Cells were then washed with Dulbecco’s Phosphate Buffer Saline (DPBS; Cat#17-515Q, Lonza, Walkersville, MD) twice and treated with 0.5mg/ml MTT. Following 3 hours of incubation at 37°C, the MTT solution was aspirated and 100μl of hydrochloride-isopropanol (0.04N) (Cat#258148, Cat#109827, Sigma-Aldrich, St. Louis, MO) was added to each well. Plates were incubated for 15 minutes on a shaker and absorbance was read at 590nm using a SpectraMax 190 microplate reader (Molecular Devices, Sunnyvale, CA).

### Determination of miR-17-3p expression level

Primary cultured macular RPE or ARPE-19 cells were seeded at 2×10^5^/ well in 6-well plates and incubated to 80% confluence. Primary cultured RPE cells were then harvested at passage 3. Total RNA was extracted from RPE cells (miRNeasy Mini kit; Cat#217004, QIAGEN, Germany). Complementary DNA (cDNA) was synthesized using predesigned TaqMan miRNA Assays (Applied Biosystems, Foster City, CA) and the Reverse Transcription Kit (Cat#4368814, Applied Biosystems, Foster City, CA) for miRNA after total RNA extraction according to the manufacturer’s instructions. Briefly, the reaction contained a volume of 5μl RNA extract, 3μl of reverse transcription (RT) primer, and 7μl master mix. RT reactions were performed on a Thermal cycler (Bio-Rad, Hercules, CA) under the following conditions: 16°C for 30 min, 42° C for 30 min, 85°C for 5 min, and 4°C on hold. All TaqMan assays were run in triplicate on an AB Step One Plus real-time PCR system using TaqMan Universal PCR Master Mix II without UNG (Cat#4440040, Applied Biosystems, Foster City, CA). Real-time PCR cycling conditions consisted of 95°C for 10 min, followed by 40 cycles of 95°C for 15 s and 60°C for 1 min.

### Western blot

Total protein was extracted from cultured ARPE-19 cells using NP40 cell lysis buffer (Cat#FNN0021, Invitrogen, Frederick, MD), and the concentration was calculated by DC protein assay (Cat#500–0016, Bio-Rad, Philadelphia, PA). Equivalent amounts of extracted protein were loaded into each well and electrophoresed on a Nupage 4–12% Bis-Tris gel (Cat#NP0335BOX, Novex, Carlsbad, CA), then transferred to a 0.2μm PVDF membrane (Cat#ISEQ00010, Millipore, Billerica, MA), blocked with non-fat dry milk (Cat#9999, Cell signaling, Danvers, MA) and incubated with primary antibodies against MnSOD (Cat#16956, Abcam, Cambridge, MA) at 1:2000, TrxR_2_ (Cat#sc-46278, Santa Cruz Biotechnology, Dallas, TX) at 1:250, and GAPDH (Cat#5174, Cell Signaling Technology, Danvers, MA) at 1:1000, for 2 hours. The membrane was washed 3 times for 10 minutes each time and incubated with secondary antibodies at 1:10000 for 1.5 hours. After washing, membranes were incubated with the chemiluminescent substrate (Cat#RPN2235, ECL Select western blotting detection reagents, GE Healthcare Life Sciences, Piscataway, NJ) for 5 minutes. Band signals were detected by an image-scanning densitometer (ChemiDoc imaging system; Bio-Rad) and quantitated by Image J 2.0.

### TUNEL (terminal deoxynucleotidyl transferase-mediated dUTP nick-end labelling) staining

ARPE-19 cells were washed with PBS once and then fixed in 4% paraformaldehyde in PBS for 10 min at room temperature (25°C). After two washes in PBS for 5 minutes each, cells were post-fixed in cooled ethanol-acetic acid (v:v, 2:1) and stained using an In Situ Apoptosis Detection Kit (Cat#S7110, Millipore, Temecula, CA) according to the manufacturer’s protocol. The cell nuclei were counterstained with DAPI (4’, 6-diamidino-2-phenylindole, Cat#62248, Life technologies). The number of TUNEL-positive cells was counted and processed for statistical analysis.

### Statistical analysis

Statistical analysis was performed on GraphPad Prism 7.0a. Statistically significant differences between two groups were assessed using Student’s t-test. One-way ANOVA followed by Tukey’s post-hoc test was applied to multiple group comparisons. Differences with *p*<0.05 were considered statistically significant.

## Results

### miR-17-3p expression increases in macular RPE cells from AMD patients

**Fig 1:**
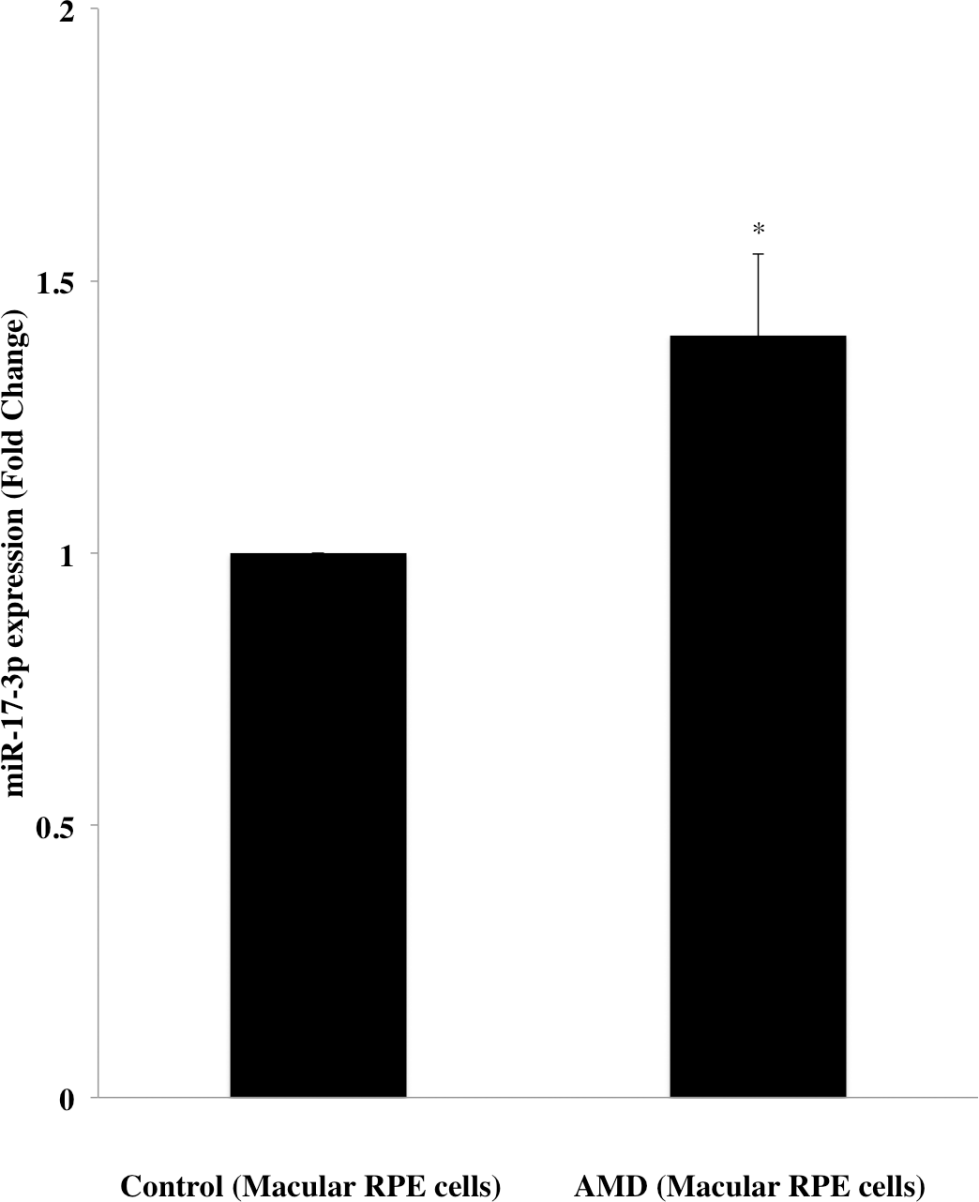
Differential miR-17-3p expression in macular RPE cells from AMD patients and normal controls. Expression levels of miR-17-3p in macular RPE cells from AMD and age-matched normal control donor eyes were analyzed by qRT-PCR. The expression levels are shown relative to control values in five independent experiments. Results are displayed as mean ± SD. *p<0.05.

A microarray assay previously performed by our group showed that miR-17-3p expression level increases in macular RPE cells of AMD patients’ donor eyes compared to age-matched normal control donor eyes. To confirm this data, total RNA was isolated from macular RPE cells of each AMD donor eye (n = 5) and age-matched normal control eye (n = 5). miR-17-3p expression level was analyzed by qRT-PCR. Fig 1 illustrates that miR-17-3p was significantly upregulated (*p*<0.05) in macular RPE cells from AMD donor eyes compared to controls (1.4-fold). It suggests that increased miR-17-3p expression may be associated with AMD.

### miR-17-3p causes ARPE-19 cell death

**Fig 2:**
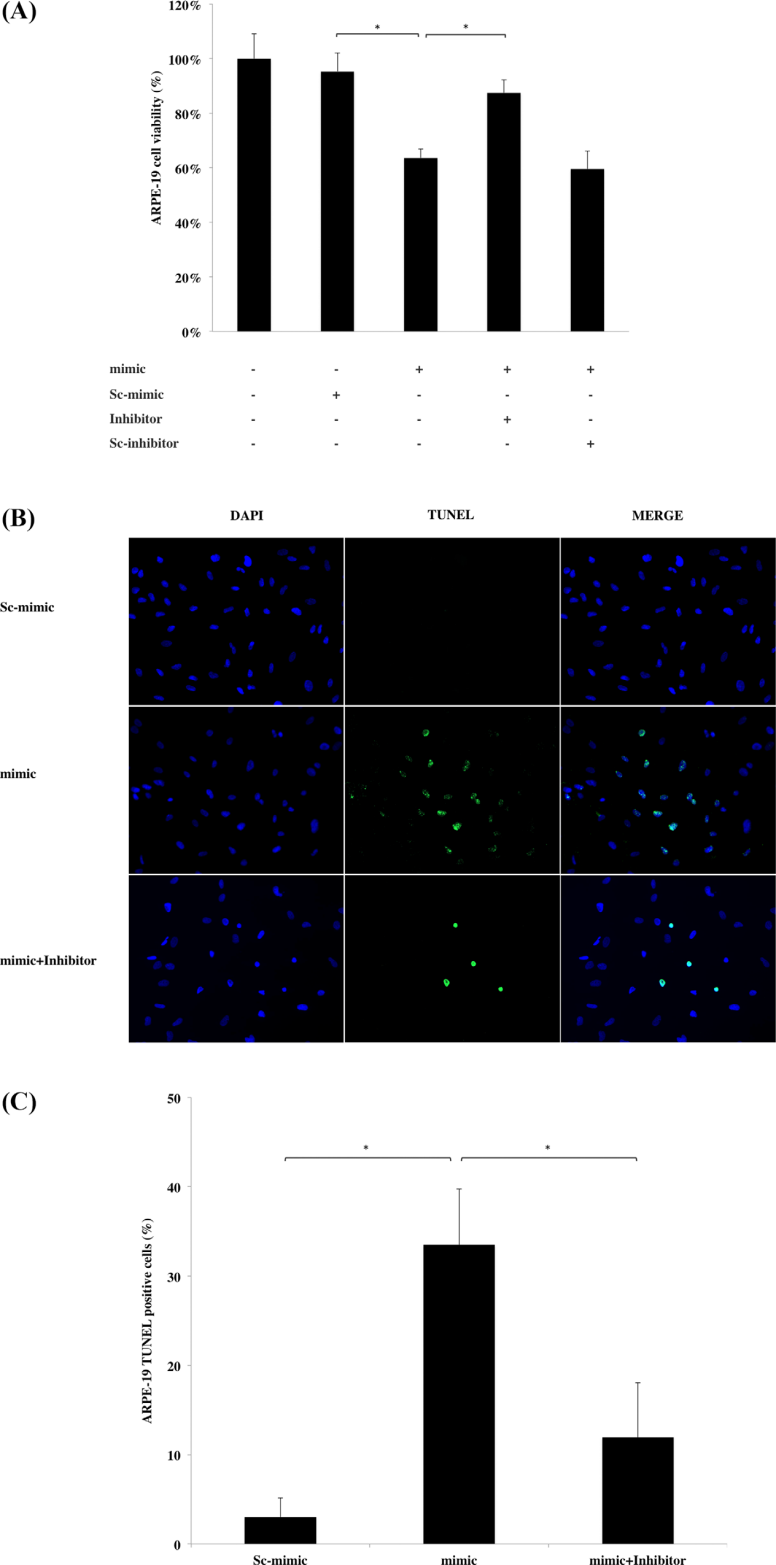
Effect of miR-17-3p on ARPE-19 cell viability. (A) ARPE-19 cells were transfected with miR-17-3p mimic (mimic), scrambled miR-17-3p mimic (sc-mimic), miR-17-3p antisense (inhibitor) or scrambled miR-17-3p antisense (sc-inhibitor) as indicated for 72 hours. Cell viability was assessed by MTT assay. The mimic sequence transfection reduced cell viability compared to scrambled sequence (sc-mimic); however, decreased cell viability was reversed by co-transfection with miR-17-3p inhibitor. Data shown as mean ± SD. (n = 8) (B) After 72 hours of transfection, cell death was evaluated by TUNEL staining. Representative images were chosen from three independent experiments. (C) The average number of TUNEL positive cells from different transfection groups was counted from six random microscope fields. Results are displayed as mean ± SD. *p<0.05.

Due to the fact that miR-17-3p was upregulated in macular RPE cells from AMD patients, we hypothesized that it may be implicated in AMD pathogenesis. Since RPE cell atrophy and death are features of AMD, we assessed cell viability by MTT assay following miR-17-3p transfection into ARPE-19 cells. miR-17-3p mimic sequence transfection caused significant reduction in cell viability (63.7%) compared to scrambled sequence (95.4%), and this effect was partially reversed by co-transfection with miR-17-3p-specific inhibitor (87.5%), but not a scrambled inhibitor (Fig 2A). Moreover, the ratio of TUNEL-positive cells/total cells in the miR-17-3p transfected group was 30.5% higher compared to the scrambled sequence group, while co-transfection with the inhibitor abrogated miR-17-3p cell death (Fig 2B and 2C). These results indicate that miR-17-3p induces ARPE-19 cell death.

### Increased expression of miR-17-3p in ARPE-19 under oxidative stress

**Fig 3:**
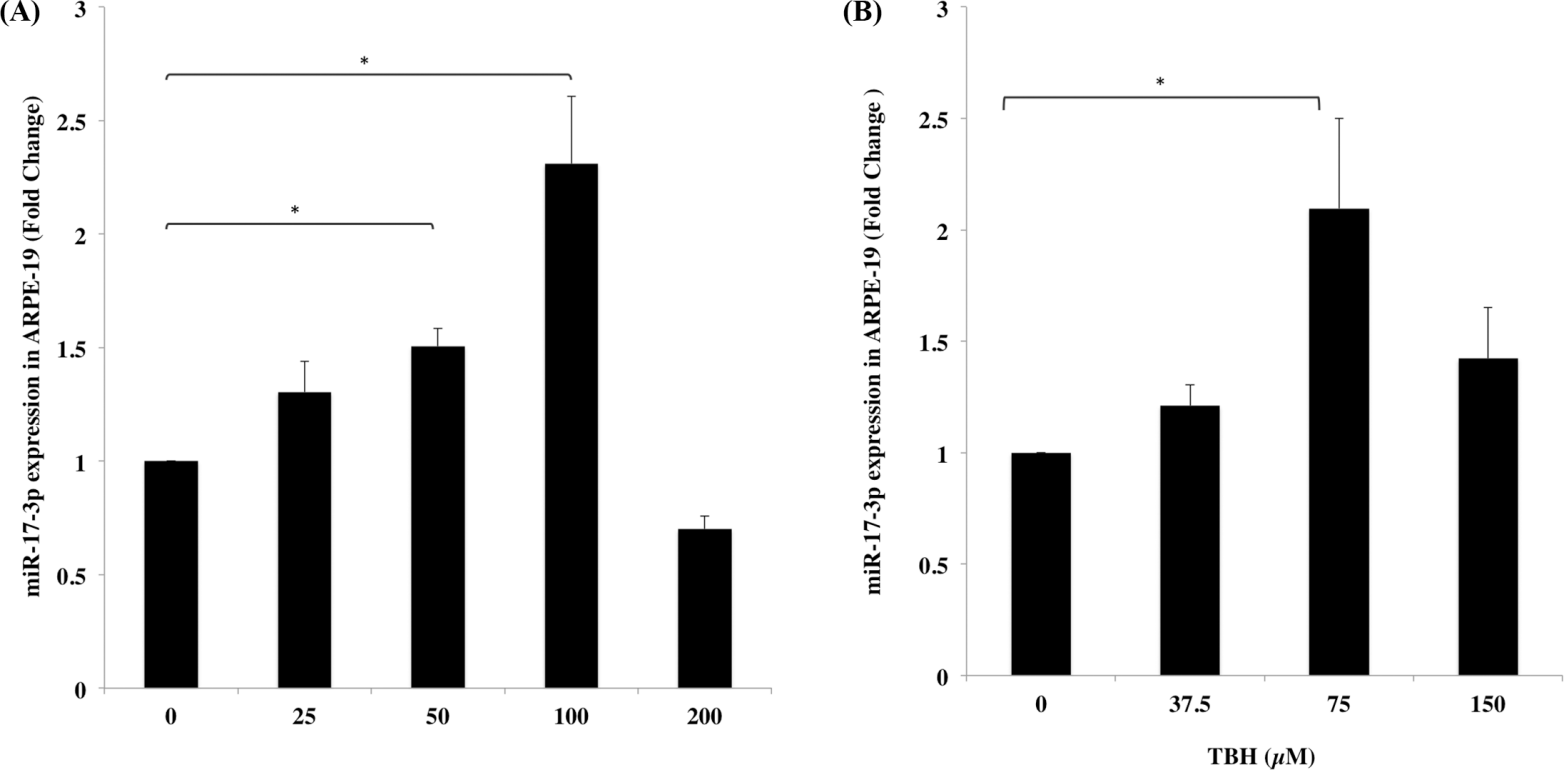
Expression of miR-17-3p in ARPE-19 cells under increasing amounts of oxidative stress. ARPE-19 cells were treated with different concentrations of H_2_O_2_ (0, 25, 50, 100, 200μM) or TBH (0, 37.5, 75, 150μM) for 6 hours. miR-17-3p expression levels were determined by qRT-PCR in both H_2_O_2_ (A) and TBH (B) treated group. Results are displayed as mean ± SD. *p<0.05.

Since oxidative stress has been linked to RPE senescence and AMD [50], we sought to evaluate miR-17-3p expression in RPE cells under oxidative stress. It was found that miR-17-3p was raised 1.3-, 1.5- and 2.3-fold in ARPE-19 cells after treatment with increasing concentrations of H_2_O_2_ (25, 50, 100μM) compared with the untreated control respectively (Fig 3A). Meanwhile, treatment with a similar oxidant stimulus, TBH, exhibited an analogous increase of miR-17-3p expression ranging from 1.2- to 2-fold compared with the untreated control (Fig 3B). However, doses higher than 100μM of H_2_O_2_ and 75μM of TBH failed to increase miR-17-3p expression, most likely due to cytotoxicity. These results indicate that miR-17-3p can be up regulated in ARPE-19 cells in the presence of oxidizing stimuli.

### miR-17-3p increases vulnerability of RPE cells to oxidative stress

**Fig 4:**
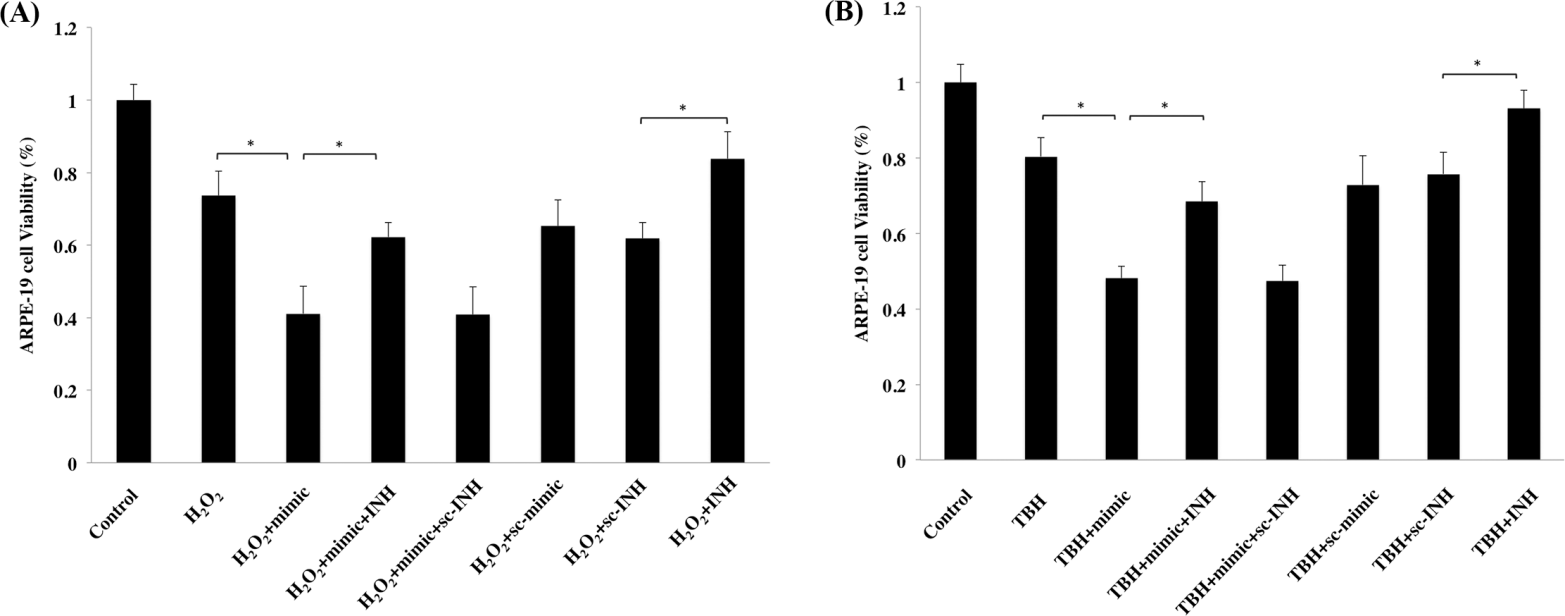
miR-17-3p increases vulnerability of RPE cells to oxidative stress. ARPE-19 cells were transfected with miR-17-3p (mimic) or miR-17-3p antisense (inhibitor, INH) as indicated for 72 hours, and then treated with (A) H_2_O_2_ (100μM) or (B) TBH (75μM) for 9 hours. Cell viability was then determined by MTT assay. Data are shown as mean ± SD. (n = 8) *p<0.05.

Since miR-17-3p was shown to cause cell death in ARPE-19 cells, and its expression was upregulated under oxidative stress, we speculated that miR-17-3p could potentially augment RPE cell death under oxidative stress. ARPE-19 cells were differentially transfected with miR-17-3p (mimic) or co-transfected with miR-17-3p antisense (inhibitor) for 72 hours, and then treated with H_2_O_2_ (100μM) or TBH (75μM) for 9 hours. Cell viability was assessed by MTT assay, which showed that cell death increased to 26.3% and 19.6% after H_2_O_2_ and TBH treatment, respectively, compared with the untreated group. Transfection with miR-17-3p mimic further increased this value to 58.9% and 51.8%, respectively (Fig 4A and 4B), whereas transfection with miR-17-3p inhibitor but not scrambled inhibitor was able to reverse H_2_O_2_ and TBH toxicity (37.7% and 31.5% of cell death by co-transfection with inhibitor in the H_2_O_2_ and TBH treated groups, respectively). Moreover, transfection with miR-17-3p antisense (INH) alone but not scrambled inhibitor (sc-INH) alone was able to partially reverse H_2_O_2_ or TBH toxicity (Fig 4). These data suggest that miR-17-3p can increase ARPE-19 cell vulnerability to oxidative stress and miR-17-3p antisense plays a protective role under oxidative stress.

### miR-17-3p reduced the expression of MnSOD and TxR_2_ in ARPE-19 cells under oxidative stress

**Fig 5:**
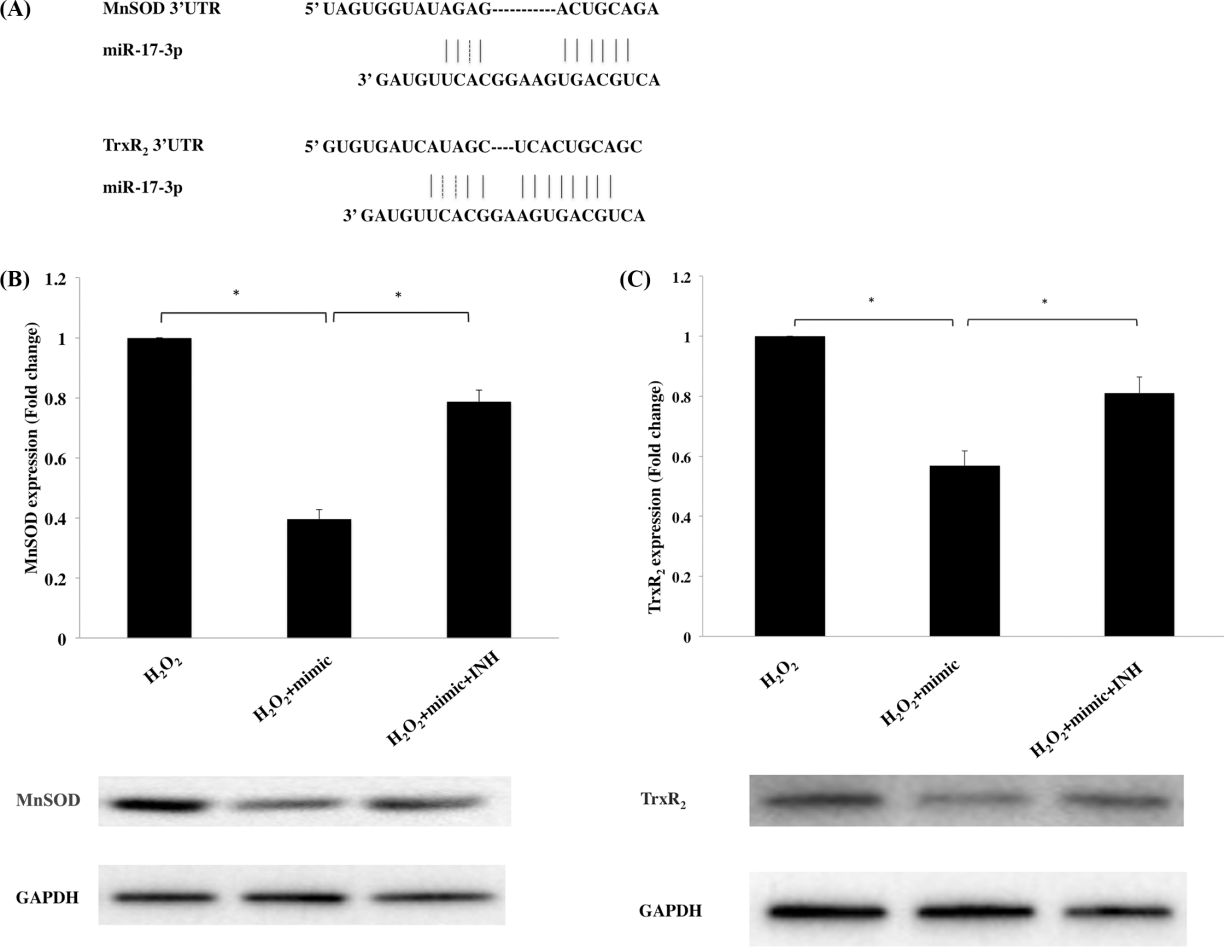
Effect of miR-17-3p on MnSOD and TrxR_2_ expression levels under oxidative stress in ARPE-19 cells. (A) Prediction of the interaction between miR-17-3p and MnSOD/TrxR_2_ mRNA 3’ UTR. ARPE-19 cells were transfected with mimic or inhibitor (INH) for 72 hours and then treated with H_2_O_2_ (100μM) for 6 hours. MnSOD (B) and TrxR_2_ (C) protein expression were assessed by Western blot analysis. Data are shown the mean ± SD from 3 independent experiments. *p<0.05

To explore the potential targets of miR-17-3p, which are responsible for the increased vulnerability under oxidative stress, we performed a bioinformatics sequence analysis and found that two antioxidant enzymes, MnSOD and TrxR_2_, possess binding sites of miR-17-3p at the 3’untranslated regions (3’ UTR) of mRNA (Fig 5A), which is validated by Xu et al. using luciferase assay [48]. miR-17-3p mimic transfection reduced MnSOD and TrxR_2_ protein expression levels to 39.6% and 56.9% respectively compared to a transfection control. Furthermore, antisense-miR-17-3p was able to reverse MnSOD and TrxR_2_ downregulations to 78.7% and 81.0%, respectively (Fig 5B and 5C). Collectively, these results demonstrate that miR-17-3p is a negative regulator of two important antioxidant enzymes in ARPE-19 cells.

## Discussion

Our study provides evidence that miR-17-3p is elevated in macular RPE cells of AMD patients and is cytotoxic to ARPE-19 cells. This cytotoxic effect can be partially reversed by the inhibitor of miR-17-3p. Furthermore, the expression level of miR-17-3p increases under oxidative stress, and it increases ARPE-19 vulnerability to oxidative stress by downregulating the mitochondrial antioxidant enzymes MnSOD and TrxR_2_. This effect can be abolished by the inhibitor of miR-17-3p.

There are only a few studies examining the role of miRNA in dry AMD. Our 2011 study [46] was the first to show that miR-23 is downregulated in human AMD eyes and its putative protective effect is mediated by downregulation of the Fas ligand. A year later, Lukiw et al. [51] showed that several miRNAs, including miRNA-9, miRNA-125b, miRNA-146a and miRNA-155, were upregulated in both Alzheimer’s disease and AMD, and they were common to the pathogenetic mechanism of complement factor H (CFH) deficiency which drives inflammatory neurodegeneration. In 2014, Murad et al. [52] reported that miR-184 was significantly downregulated in primary RPE cells isolated from AMD donors, and its downregulation resulted in impaired autophagy and dysfunction of the RPE. More recently, Szemraj et al. [53] showed that there is an altered serum profile of miRNA in wet versus dry AMD patients, suggesting a potential role for miRNAs as novel biomarkers. Our current study shows that miR-17-3p not only is upregulated in the RPE cells of AMD patients, but it also increases vulnerability to oxidative stress in vitro and causes ARPE-19 death itself, suggesting that miR-17-3p may be contributing to the reduced RPE viability caused by oxidative stress in AMD patients.

RPE degeneration in AMD has been strongly linked to oxidative stress [54]. Oxidative stress can be induced by exposing of cells to oxidant generators, such as H_2_O_2_ or TBH. H_2_O_2_, one of the major free radicals and a precursor of highly oxidizing, tissue-damaging radicals, has been known to cause RPE damage and cell death [55]. Moreover, H_2_O_2_ triggers changes in the expression of multiple genes, which are involved in ROS-mediated RPE cell death and apoptosis [46,55,56]. TBH is a short-chain organic hydro peroxide, also used as an oxidant for investigating mechanisms of cellular alterations resulting from oxidative stress in cells and tissues, such as the RPE [57]. We found that miR-17-3p was elevated in ARPE-19 cells after treatment with increasing concentrations of oxidizing reagents H_2_O_2_ and TBH. Multiple oxidation-sensitive genes and factors are induced in RPE cells exposed to ROS [58,59]. Alterations in gene expression in response to oxidative stress have been extensively studied at transcriptional levels [60,61], however it is accepted that post-transcriptional regulation of gene expression is also very important [62]. MicroRNAs serve as significant post-transcriptional regulators of gene expression.

Evidence stemming from prior studies in prostate cancer cells as well as from our bioinformatic analysis suggests that antioxidant enzymes MnSOD and TrxR_2_ act as targets of miR-17-3p [48]. MnSOD, a ROS scavenger, plays a vital role in promoting cell survival under stressful conditions, and several studies have confirmed that the decrease in cell viability observed under oxidative stress is correlated with MnSOD downregulation [21,63]. Moreover, some exogenous antioxidants, such as estrogen and progesterone, exhibit their antioxidant function by increasing the expression of mitochondrial-localized gene products, such as MnSOD and Bcl-2, which display antioxidant activities [64–66]. Verline et al. found that MnSOD knockdown in mice leads to oxidative damage of RPE cells, and more importantly, the appearance of certain key features of AMD [67]. The mitochondrial enzyme TrxR_2_ preserves thioredoxin in a reduced state, thereby playing a key role in maintaining the cellular redox environment [68]. Inhibition of TrxR_2_ in mitochondria isolated from guinea pigs, as well as in mouse cardiomyocytes, resulted in increased H_2_O_2_ production [24]. This correlates with our findings, as cell viability was reduced with miR-17-3p mimic transfection under oxidative stress, in parallel with MnSOD and TrxR_2_ downregulation.

Due to increased miR-17-3p levels in AMD eyes in combination with the fact that miR-17-3p can specifically downregulate MnSOD and TrxR_2_ expression, its role as a mediator for RPE susceptibility to oxidative stress in AMD should not be overlooked. Interestingly, another recent study by Haque et al. [43] showed that in ARPE-19 cells, a sublethal dose of H_2_O_2_ upregulated miR-30b, which inhibited catalase, another anti-oxidant enzyme. Furthermore, a miR-30b antagonist protected RPE cells from oxidative stress, concurring with our findings after using miR-17-3p.

miR-17-3p is a member of miR-17/92 cluster which is extensively studied in tumorigenesis [69]. Most of the work on miR17-3p has thus been focused on studying the regulation of cell proliferation pathways. More recently though, studies have shown more varied effects of miR-17-3p, such as involvement in development and spinal progenitor cells [70], induction by TNF alpha and LPS in HeLa cells [71], downregulating flk-1 in endothelial cells [72], suppressing epithelial to mesenchymal transformation of ovarian epithelial cells [73], inhibiting fibroblast senescence [74] and being downregulated in the plasma of Alzheimer patients [75]. More relevant to our work, it has been shown that miR-17-3p downregulates important anti-oxidant enzymes, such as manganese superoxide dismutase, glutathione peroxidase-2 and thioredoxin reductase-2 (TrxR_2_) in prostate cancer cell lines [48] and blood mononuclear cells [76]. To confirm the previous studies, our bioinformatic sequence analysis found that there is a putative binding site for the miR-17-3p in the mRNA 3’untranslated regions of MnSOD and TrxR_2_ (Fig 5A) that is verified by in vitro experiments (Fig 5B and 5C). The mechanism by which oxidative stress regulates miR-17-3p levels remains elusive. It is interesting to note that downregulation of c-Myc suppresses pri-miR-17/92 cluster expression [77] and that c-Myc is downregulated by peroxiredoxin 1, which is involved under oxidative stress [78]. Although this mechanism has not been studied in RPE cells or in AMD, it is a promising target for future investigation.

In conclusion, this study demonstrates increased miR-17-3p expression in human AMD eyes. It also shows that miR-17-3p expression is increased by oxidative stimuli and that it has detrimental effects on RPE antioxidant defense mechanisms by downregulating the anti-oxidant enzymes MnSOD and TrxR_2_. Therefore, inhibition of miR-17-3p could be a novel therapeutic approach to protect RPE cells from oxidative stress.
